# Effects of Dietary Dandelion Supplementation on Ruminal Morphology, Fermentation, Microbiome, and Inflammation in Lambs Under High-Concentrate Feeding

**DOI:** 10.3390/ani16162585

**Published:** 2026-08-19

**Authors:** Yue Zheng, Wenwen Wang, Huihui Yang, Yuan Wang, Tao Guo, Na Yin, Hongfei Liang, Baishan Song, Yang Jia, Ruixue Nie, Yaguang Zheng, Ruiguang Gong, Jingwei Qi

**Affiliations:** 1 College of Animal Science, Inner Mongolia Agricultural University, Hohhot 010018, China; 2 Dairy-Breeding and Production Research Subcenter of National Technology Innovation Center for Dairy, Hohhot 010018, China; 3 Key Laboratory of Smart Animal Husbandry at Universities of Inner Mongolia Automomous Region, Hohhot 010018, China

**Keywords:** lambs, high-concentrate diet, dandelion, ruminal inflammatory damage, metagenomics

## Abstract

This study aimed to investigate the regulatory effects of dietary dandelion supplementation on the ruminal physiological status of lambs fed a high-concentrate diet. Twenty-two 6-month-old female crossbred lambs with an initial body weight of 32.05 ± 0.43 kg were randomly assigned to two groups (n = 11 per group): the HC group (fed a high-concentrate basal diet) and the DD group (basal diet supplemented with 2 g/kg dandelion powder), with a 14-day adaptation and 60-day experimental period. The results showed that compared with the HC group, dandelion supplementation significantly improved lamb growth performance and modulated ruminal fermentation parameters and tissue morphology. For inflammatory indices, the concentration of interleukin-10 (IL-10) was significantly increased, while the concentrations of interleukin-1β (IL-1β), lipopolysaccharide (LPS) and histamine (HIS) were significantly decreased (*p* < 0.05). Meanwhile, dandelion supplementation upregulated the mRNA expression of ruminal epithelial tight junction proteins and hexokinase II (HK-II), while downregulating the mRNA expression of myeloid differentiation factor 88 (MyD88), voltage-dependent anion channel 1 (VDAC1), inositol 1,4,5-trisphosphate receptor (IP3R), NLR family pyrin domain containing 3 (NLRP3), and caspase-1. Metagenomic analysis revealed that dandelion supplementation did not alter ruminal microbial α-diversity, but slightly modulated community composition and functional profiles. The abundances of dominant phyla Bacteroidota and Bacillota showed only slight fluctuations, while fiber-degrading genera (*Xylanibacter*, *Quinella*, *Selenomonas*) showed a trend toward enrichment, and proteolytic taxa were decreased. CAZyme analysis revealed an upward trend in families CE4, CE8, GH32, and GH13_46 (pectin/starch degradation) and a downward trend in GH73, GH27, and GT14 (oligosaccharide/peptidoglycan/polysaccharide metabolism) in the DD group. KEGG orthology annotation suggested that nominal KO differences were mainly associated with carbohydrate metabolism pathways, including starch and sucrose metabolism and peptidoglycan biosynthesis. None of these features, however, remained significant after FDR correction (raw *p* < 0.05; all *q* > 0.05). Collectively, dietary dandelion supplementation was associated with alterations in ruminal physiological status and microbial community in lambs fed a high-concentrate diet, providing foundational data for dandelion application in lamb diets under short-term feeding conditions.

## Simple Summary

Long-term feeding of high-concentrate diets commonly induces ruminal inflammatory injury in fattening lambs. This study investigated the regulatory effects of dietary dandelion supplementation on ruminal status in lambs under a high-concentrate feeding regime. Compared with the high-concentrate control group, dietary dandelion supplementation improved growth performance, modulated ruminal morphology and fermentation parameters, enhanced ruminal epithelial tight junction integrity, reduced concentrations of inflammatory factors, and concurrently downregulated the mRNA expression of inflammatory genes. Metagenomic analysis revealed that dandelion supplementation did not alter ruminal microbial α-diversity, but slightly modified community composition and functional profiles. Specifically, the abundances of fiber-degrading taxa were increased, whereas those of proteolytic taxa were decreased. Additionally, trends were observed in CAZymes and KEGG functional genes, although these did not reach statistical significance after correction for multiple comparisons. Collectively, these findings provide foundational data for the application of dandelion in high-concentrate lamb diets under short-term feeding conditions, while its long-term effects warrant further investigation.

## 1. Introduction

In intensive livestock farming systems, high-concentrate diets are widely used to improve the fattening efficiency of lambs and meet the rapidly growing demand for meat production [1]. However, excessive concentrate intake inevitably leads to a rapid decline in rumen pH in ruminants, which can easily induce subacute ruminal acidosis, disrupt ruminal fermentation homeostasis, and consequently impair the ruminal epithelial barrier function and trigger inflammatory responses [2]. This ultimately results in digestive and metabolic disorders and stunted growth in lambs, causing economic losses to the mutton sheep industry. Furthermore, high-concentrate diets can induce oxidative stress, impair host immune function, and adversely affect meat quality characteristics [3]. Therefore, alleviating ruminal inflammatory damage and disruption of ruminal homeostasis induced by high-concentrate diets is essential not only for promoting efficient and healthy mutton sheep production but also for sustaining the health and optimal production performance of fattening lambs. Plant-based feed additives, characterized by their safety, absence of residues, and multiple physiological functions such as antioxidation, anti-inflammation, and regulation of rumen fermentation, have significant application value in improving metabolic disorders caused by high-concentrate diets and maintaining rumen health in ruminants [4]. However, the biological activities of phytogenic feed additives are determined by their complex chemical constituents. Distinct bioactive metabolites unique to different plant species mediate disparate molecular functional targets, which hinders the establishment of universal regulatory frameworks [5].

Among various herbal alternatives, dandelion has attracted extensive research attention for ruminant feeding. Dandelion (*Taraxacum mongolicum Hand.-Mazz.*), a perennial Asteraceous herb from the genus *Taraxacum*, is widely distributed throughout the world and is particularly common in grasslands, fields and roadsides across Asia, Europe and North America [6]. As a well-known medicinal plant, the main active compounds in dandelion include polysaccharides, flavonoids and phenolic acids; such constituents exhibit a range of biological activities, mainly including antioxidant and anti-inflammatory properties [7]. Wang et al. performed a study using lactating Holstein cows fed high-concentrate diets. Their data revealed that excessive concentrate intake reduced ruminal pH and elevated circulating LPS, triggering systemic oxidative stress and impairing mammary gland health. Supplementation with 0.5% dandelion aqueous extract restored ruminal pH, lowered endotoxin concentrations in blood and milk, and relieved oxidative injury by activating the Nrf2 antioxidant pathway, thereby sustaining milk yield while alleviating ruminal and mammary damage induced by high-grain feeding [8]. Mgeni et al. supplemented 1% dandelion extract to high-concentrate diets for dairy goats. High-concentrate feeding decreased ruminal pH and disturbed ruminal fermentation homeostasis, whereas dandelion extract supplementation moderately elevated ruminal pH and markedly increased acetate concentration and serum IgA to enhance immune function [7]. These two studies analysed the anti-inflammatory protective effects of dandelion from different analytical perspectives: one focuses on rumen microbial composition and the host’s immune phenotype, while the other elucidates the underlying mechanisms through the antioxidant signalling pathways in the mammary gland. Among these, the research concerning the rumen microbiota is limited to analysing differences in species abundance and does not further explore the functional gene information of rumen microorganisms. Furthermore, most existing studies have utilised dandelion extracts as the test material, whilst research data on the application of whole-plant dandelion powder in ruminant diets remains relatively limited.

Although dandelion has been reported to exert beneficial effects on ruminal function in ruminants and anti-inflammatory activity, systematic investigations into its regulatory roles in ruminal microbiota, fermentation and inflammatory status in fattening lambs under high-concentrate feeding remain very limited. Furthermore, existing studies on the microbiome are confined to analyses of taxonomic composition, lacking an examination of the functional characteristics of rumen microbiota. Accordingly, this study aims to systematically investigate the effects of dandelion supplementation on growth performance, rumen morphology, fermentation parameters, inflammatory status, and the structure and function of the rumen microbiome in lambs fed a high-concentrate diet, by integrating physiological parameter determination, inflammation-related gene transcription analysis and metagenomic sequencing. In summary, our study proposed two hypotheses: (1) Dietary addition of dandelion can improve growth performance, optimize rumen fermentation patterns, and improve ruminal epithelial morphological status in lambs fed high-concentrate diets. (2) Dandelion can reshape the classification and functional characteristics of rumen microflora and regulate the transcription of inflammation-related genes, thereby further enhancing rumen barrier function and modulating ruminal inflammatory responses under high-concentrate feeding conditions.

## 2. Materials and Methods

### 2.1. Animal Ethics Statement

The approval of the animal experiment protocol for this study was provided by the Animal Use and Care Committee of Inner Mongolia Agricultural University (NND2024032). All animal experiment protocols strictly followed the requirements of the ethical procedures and guidelines of the People′s Republic of China.

### 2.2. Dandelion

The entire dandelion was harvested from June to July and purchased from Hohhot, Inner Mongolia, China. Fresh raw materials were shade-dried at ambient temperature, then ground and sieved through a 60-mesh screen. The processed dandelion powder was stored in a dry environment at room temperature prior to diet formulation. The dandelion contained flavonoids, polyphenols, and polysaccharides at levels of 35.00, 43.48, and 193.93 mg/g, respectively. The flavonoid content was assayed by the aluminum salt colorimetric method, whose standard curve was y = 3.3159x + 0.035 (*R*^2^ = 0.999) [9]. The polyphenol content was measured via the Folin–Ciocalteu method, and its standard curve was y = 0.5721x + 0.06 (*R*^2^ = 0.9988) [10]. The polysaccharide content was determined using the phenol-sulfuric acid method, with the standard curve equation y = 3.3714x + 0.1876 (*R*^2^ = 0.9976) [11].

### 2.3. Experimental Design and Dietary Formulation

A total of twenty-two 6-month-old female Dumeng crossbred lambs (Dorper × Mongolian sheep) with uniform initial body weight (32.05 ± 0.43 kg) were randomly allocated to two treatment groups, each containing 11 lambs. All lambs received a high-concentrate basal diet; animals in the high-concentrate control group (HC) were offered only the basal ration, whereas the dandelion treatment group (DD) was supplied with the identical basal diet supplemented with 2 g/kg dandelion powder of feed. This supplemental dosage was selected in reference to our prior in vitro experimental, which confirmed that the 2 g/kg dandelion inclusion level could optimize ruminal fermentation profiles. The entire feeding experimental lasted for 74 days in total, consisting of a 14-day preliminary adaptation phase followed by a 60-day formal experimental period. Table 1 lists all raw material proportions and corresponding nutritional parameters of the basal experimental diet. All experimental lambs were raised in independent pens inside a naturally ventilated windowed barn, equipped with separate feed troughs and drinking water supplies for individual feeding. Daily feeding was implemented at 08:00 and 17:00, and clean fresh water was available ad libitum to all lambs throughout the entire trial.

### 2.4. Growth Performance

The weight of feed offered to each lamb was weighed and recorded daily prior to the morning feeding. Residual feed in each feed trough was fully collected and weighed at a fixed time every morning. Average daily feed intake (ADFI) was calculated as the mean of the daily difference between offered feed weight and residual feed weight throughout the formal experimental period. Initial body weight (Initial BW) was recorded on the morning of day 0, which marked the onset of the formal experiment following the 14-day adaptation phase. Final body weight (Final BW) was measured on the morning of day 60. Prior to each weighing, all lambs underwent a 12 h overnight feed deprivation with ad libitum access to clean water to ensure a consistent fasting status. Average daily gain (ADG) was computed as the difference between Final BW and Initial BW divided by the total number of days in the formal experimental period. The feed-to-gain ratio (F:G ratio) was further derived from the ratio of ADFI to ADG, calculated for each lamb individually and then averaged within group.

### 2.5. Ruminal Sample Collection

Following body-weight measurement, all lambs were given a small amount of diet and then subjected to a 2 h fasting period with ad libitum access to water prior to slaughter. To minimize alterations in microbial activity and ruminal fermentation profiles, all rumen samples were processed within 25 min following slaughter. For metagenomic sequencing of the ruminal microbiota, whole ruminal digesta samples from each lamb were individually transferred into 2.5 mL sterile polypropylene cryotubes, immediately snap-frozen in liquid nitrogen, and subsequently stored at −80 °C until further analysis. For ruminal fermentation parameter analysis, approximately 100 mL of whole ruminal fluid was collected per lamb and filtered through four layers of sterile cheesecloth. In addition, approximately 15 g of ruminal tissue samples were excised, placed into 5 mL sterile polypropylene cryotubes, immediately immersed in liquid nitrogen, and stored at −80 °C for subsequent enzyme-linked immunosorbent assay (ELISA) and quantitative real-time polymerase chain reaction (qRT-PCR) analyses. For histological evaluation, ruminal tissue specimens (1.5 cm × 1.5 cm in dimension) were collected and fixed in 4% (*w*/*v*) paraformaldehyde solution. All samples were kept at room temperature in the dark and then processed for paraffin embedding, serial sectioning, and hematoxylin and eosin staining for morphological observation.

### 2.6. Ruminal Epithelial Histomorphology

The morphological indicators of the rumen epithelium were determined according to the previously validated histological method [8]. The specific operation procedure is as follows: The fixed rumen tissue specimens were dehydrated with gradient ethanol, embedded in paraffin, and sectioned continuously. The prepared sections were stained with hematoxylin–eosin (H&E), and then observed under a Motic BA610(Motic (Xiamen) Electric Group Co., Ltd., Xiamen, China) optical microscope equipped with a digital imaging sensor. Quantitative analysis of six morphological indicators—epithelial cell layer thickness, basal membrane thickness, lamina propria thickness, muscular layer thickness, papillary length, and papillary width—was conducted. Image acquisition and parameter quantification were completed using the Motic DS Assistant Lite image analysis software (VM V1 Viewer 1.0).

### 2.7. Ruminal Fermentation Parameters

Immediately after filtering ruminal liquid, pH values were quantified via a calibrated PHS-3C portable pH detector (Puchun Metrology Instrument Co., Ltd., Shanghai, China). To test ammonia nitrogen (NH_3_-N) levels, 0.5 mL filtrate was mixed thoroughly with 4 mL of 0.2 mol/L hydrochloric acid solution, and the mixed sample was assayed by standard colorimetric procedures [12]. All individual VFA components were quantified by gas chromatography after preconditioning 4 mL filtrate with 1 mL metaphosphoric acid [13]. The total amino acid concentration was determined using a total amino acid assay kit (Bioscience Engineering, Nanjing, China).

### 2.8. Ruminal Tissue Cytokine Determination

Approximately 1 g of ruminal tissue was homogenized with normal saline at a mass-to-volume ratio of 1:9. After full tissue lysis and mixing, the mixture underwent centrifugation, and the resulting supernatant was collected as the test sample. The concentrations of interleukin-1β (IL-1β), interleukin-6 (IL-6), interleukin-10 (IL-10), tumor necrosis factor-α (TNF-α), lipopolysaccharide (LPS) and histamine (HIS) were measured using commercial ELISA kits supplied by Wuhan Colorful Gene Biological Technology Co., Ltd., Wuhan, China.

### 2.9. Quantitative Real-Time PCR Analysis

Total RNA was extracted from rumen tissue using TRIzol Reagent (Invitrogen, Carlsbad, CA, USA). RNA purity and integrity were assessed using a DU 640 UV spectrophotometer (Beckman Coulter, Brea, CA, USA) and 1% agarose gel electrophoresis, respectively; all samples had OD260/OD280 ratios between 1.8 and 2.0. cDNA was synthesized using the SuperScript III First-Strand Synthesis System (Invitrogen, USA). Quantitative real-time PCR was performed on a LightCycler 480 System (Roche, Basel, Switzerland) with SYBR Green Master Mix (Roche, Basel, Switzerland), using β-actin as the internal reference gene (CV = 4.13%). The thermal cycling protocol consisted of an initial denaturation at 95 °C for 1 min, followed by 40 cycles of 95 °C for 15 s and 63 °C for 25 s. Relative mRNA expression was calculated using the 2-ΔΔCt method and normalized to the HC group. Primer sequences are provided in Table 2.

### 2.10. Rumen Microbiome Metagenomics Analysis

Total microbial genomic DNA was extracted from sheep ruminal digesta using the Mag-Bind Soil DNA Kit (Omega Bio-tek, Norcross, GA, USA). DNA concentration, purity and integrity were evaluated with a NanoDrop 2000 spectrophotometer (Thermo Fisher Scientific, Waltham, MA, USA) and 1% agarose gel electrophoresis. Qualified DNA was fragmented to approximately 350 bp fragments by a Covaris M220 sonicator (Covaris, Woburn, MA, USA), and paired-end metagenomic libraries were constructed with the NEXTFLEX Rapid DNA-Seq kit (Revvity, Ann Arbor, MI, USA). Whole-genome shotgun sequencing was conducted on the Illumina NovaSeq 6000 platform(Illumina, San Diego, CA, USA). Raw reads were quality controlled by fastp v0.23.0 to remove adapters, ambiguous sequences, and low-quality reads with average Q-value < 20 or length < 50 bp. Host sheep-derived reads were filtered out by mapping clean reads to the ovine reference genome via BWA v0.7.9a. The remaining microbial reads were de novo assembled into contigs (≥300 bp) using MEGAHIT v1.1.2. Open reading frames (ORFs) longer than 100 bp were predicted by Prodigal (Hyatt et al., Knoxville, TN, USA) and translated into amino acid sequences. A non-redundant gene catalog was constructed with CD-HIT (Li & Godzik, JCVI, La Jolla, CA, USA) at 90% identity and coverage, and gene abundance was quantified using SOAPaligner v2.21. Functional and taxonomic annotation was performed with Diamond v0.8.35 (e-value ≤ 10^−5^): the NR database was used for taxonomic classification of rumen microbes; the KEGG database was adopted to characterize microbial metabolic pathways related to nutrient degradation and cellular signaling; the CAZy database was annotated to identify six families of carbohydrate-active enzymes. The Benjamini–Hochberg false discovery rate (FDR) procedure was applied to correct for multiple comparisons across all tested features. An adjusted *q*-value < 0.05 was considered statistically significant. All bioinformatic analyses were completed on the Majorbio Cloud Platform (Macrogenomics 2.0, Shanghai Majorbio Bio-pharm Technology Co., Ltd., China). The obtained raw paired-end reads were deposited in the NCBI Sequence Read Archive (BioProject ID: PRJNA1479742).

### 2.11. Statistical Analysis

Data were analyzed using SAS 9.2 (SAS Institute, Cary, NC, USA) in a completely randomized design, with individual lamb as the experimental unit. Growth performance, ruminal fermentation parameters, ELISA, and qPCR data were analyzed using *t*-test, and results are presented as means ± SEM. Pearson correlation analysis was performed to examine the relationships among inflammatory mediators, with significance set at *p* < 0.05. Differential analysis of microbial taxa, CAZymes, and KEGG orthologs between groups was performed using the Wilcoxon rank-sum test, with results expressed as mean ± SD. Beta diversity analysis based on Bray–Curtis distance metrics was conducted to assess microbial community variation across samples, visualized via principal coordinate analysis (PCoA), with statistical significance evaluated by PERMANOVA (Adonis test; *p* < 0.05).

## 3. Results

### 3.1. Growth Performance

Table 3 displays the effects of adding DD to the diet on the growth performance of lambs. As shown in the table, the initial body weights of the two groups of lambs were similar (*p* > 0.05). Following the addition of dandelion to the diet, the final body weight, ADG and ADFI of the DD group were significantly higher than those of the HC group (*p* < 0.05). However, there was no significant difference in the feed conversion ratio between the two groups (*p* > 0.05).

### 3.2. Rumen Morphology

**Figure 1:**
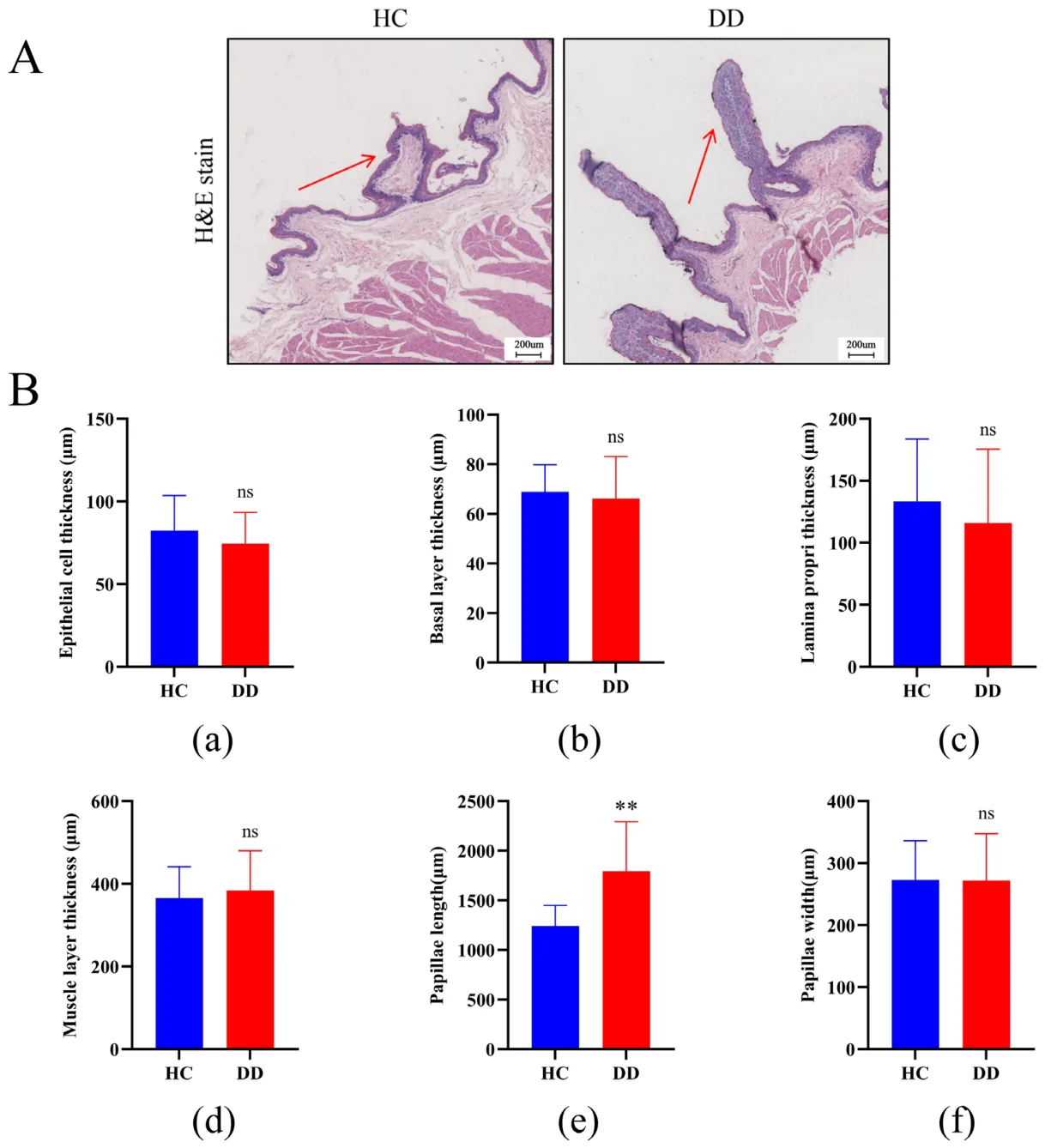
Effects of dietary DD supplementation on the rumen morphology of lambs. (**A**) HE-stained sections of rumen tissue. (**B**) Conventional morphological parameters. (**a**) Thickness of the epithelial cell layer. (**b**) Thickness of the basal lamina. (**c**) Thickness of the lamina propria. (**d**) Thickness of the muscular layer. (**e**) Length of the papillae. (**f**) Width of the papillae. Data are expressed as mean ± SEM. Significant differences are indicated as ** *p* ≤ 0.01, ns *p* > 0.05. Scale bar = 200 μm.

Compared with the HC group, the DD group exhibited marked morphological changes in the rumen of sheep (Figure 1A), with rumen papillae in the DD group being significantly longer than those in the HC group (*p* < 0.01). However, there were no significant differences between the two groups in terms of the thickness of the epithelium, basal layer, lamina propria, and muscular layer, or in papillae width (*p* > 0.05, Figure 1B).

### 3.3. Ruminal Fermentation Parameters

The Ruminal Fermentation Parameters are shown in Table 4. Compared with the HC group, there were no significant differences in ruminal pH and NH_3_-N concentration in the DD group (*p* > 0.05); however, the levels of total amino acids and total VFA in the DD group were significantly higher than those in the HC group (*p* < 0.05). Furthermore, the proportions of Acetate, Propionate and Valerate in the DD group were significantly higher than those in the HC group (*p* < 0.05), whilst there were no significant differences in the proportions of isobutyrate and isovalerate (*p* > 0.05). There was no statistically significant difference in the acetate-to-propionate ratio (Acetate: Propionate) between the two groups (*p* > 0.05).

### 3.4. Ruminal Inflammatory and Abnormal Metabolite Indices

**Figure 2:**
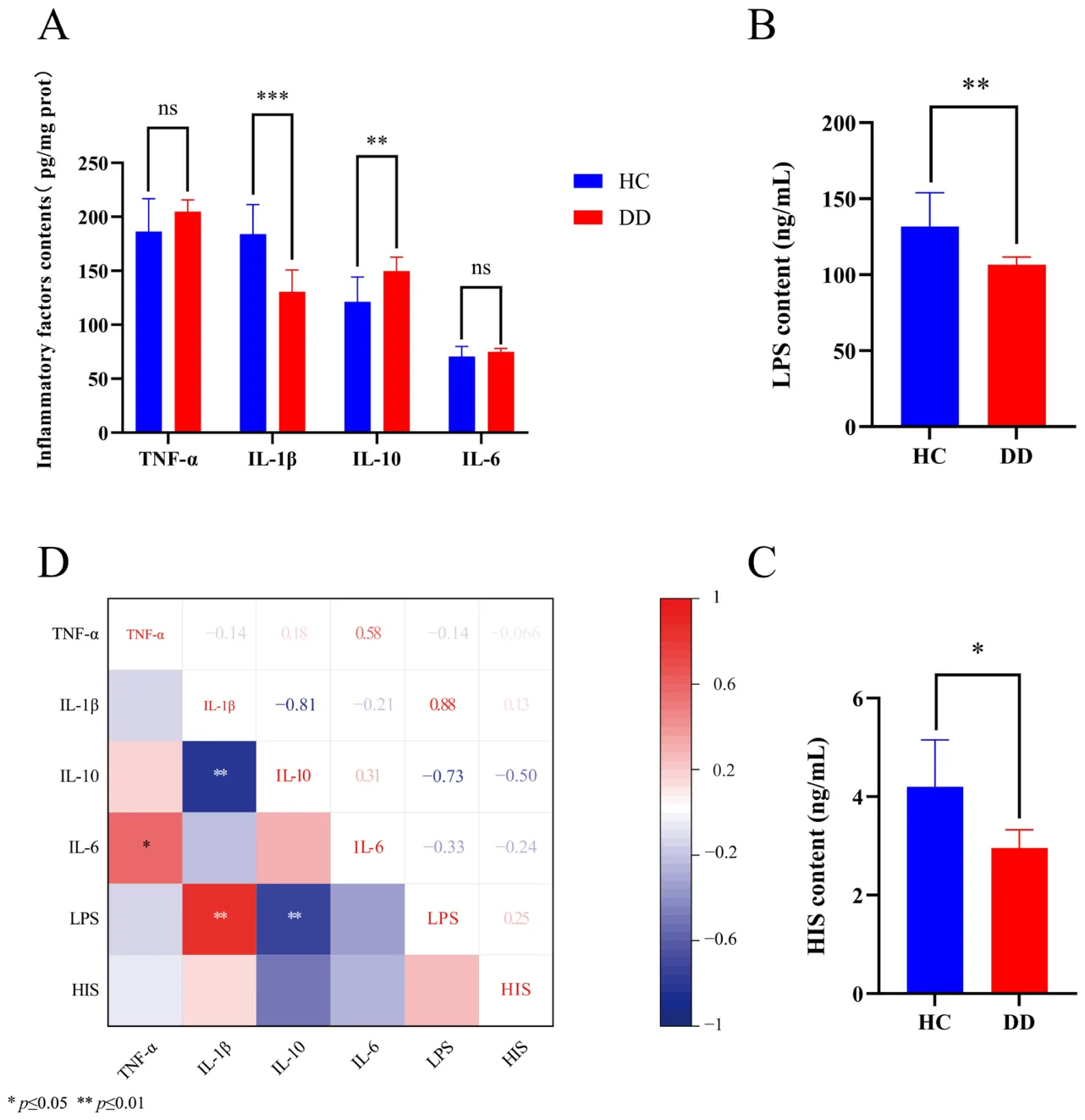
Effects of dietary DD supplementation on levels of immune and inflammation-related markers in the rumen of lambs. (**A**) Levels of TNF-α, IL-1β, IL-10 and IL-6. (**B**) LPS levels. (**C**) HIS levels. (**D**) Correlation analysis. Data are mean ± SEM, n = 11 per group. Two-group comparisons were performed using an unpaired *t*-test. * *p* ≤ 0.05, ** *p* ≤ 0.01, *** *p* ≤ 0.001, ns *p* > 0.05.

Compared with the HC group, the concentration of IL-1β was significantly decreased and the concentration of IL-10 was significantly increased in the DD group (*p* < 0.05, Figure 2A). However, the concentration of TNF-α and IL-6 was not significantly affected by DD treatment (*p* > 0.05, Figure 2A). More notably, DD treatment significantly reduced the contents of LPS and HIS (*p* < 0.05, Figure 2B,C). Pearson′s correlation analysis of inflammatory mediators found that IL-1β exhibited an extremely significant positive correlation with LPS (r = 0.88, *p* < 0.01), whereas IL-1β was strongly and negatively correlated with anti-inflammatory IL-10 (r = −0.81, *p* < 0.01). Meanwhile, IL-10 showed an extremely significant negative association with LPS (r = −0.73, *p* < 0.01). A moderately significant positive correlation was observed between two pro-inflammatory cytokines TNF-α and IL-6 (r = 0.58, *p* < 0.05). No statistically meaningful correlations were detected between HIS and all detected inflammatory factors (Figure 2D).

### 3.5. Tight Junction Proteins and HK-II/NLRP3 Signal Pathway

**Figure 3:**
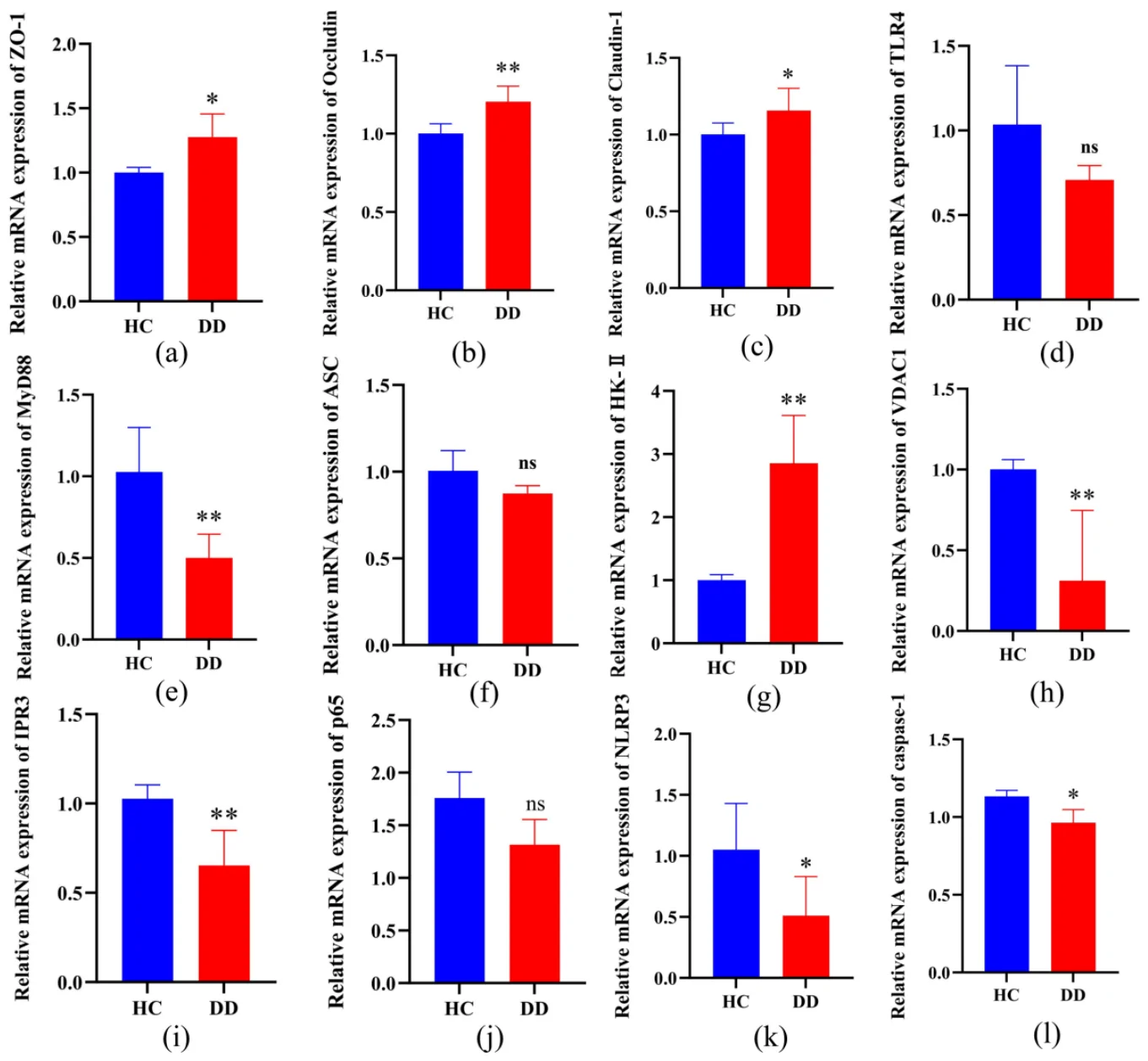
Effects of dietary DD supplementation on the ruminal barrier and the expression of genes associated with inflammatory pathways in lambs. (**a**) ZO-1; (**b**) Occludin; (**c**) Claudin-1; (**d**) TLR4; (**e**) MyD88; (**f**) ASC; (**g**) HK-II; (**h**) VDAC1; (**i**) IP3R; (**j**) p65; (**k**) NLRP3; (**l**) caspase-1. Data are mean ± SEM, n = 8 per group. Two-group comparisons were performed using an unpaired *t*-test. * *p* ≤ 0.05, ** *p* ≤ 0.01, ns *p* > 0.05.

Compared with the HC group, the mRNA expression levels of the ZO-1, Occludin, Claudin-1, and HK-II genes were all significantly upregulated in the rumens of sheep fed DD (*p* < 0.05, Figure 3). In contrast, the mRNA expression levels of the MyD88, VDAC1, IP3R, NLRP3 and caspase-1 genes were significantly downregulated in the rumens of sheep in the experimental group (*p* < 0.05). Additionally, no significant differences were observed in the expression levels of TLR4, ASC and p65 (*p* > 0.05).

### 3.6. Rumen Microbiome Metagenome

**Figure 4:**
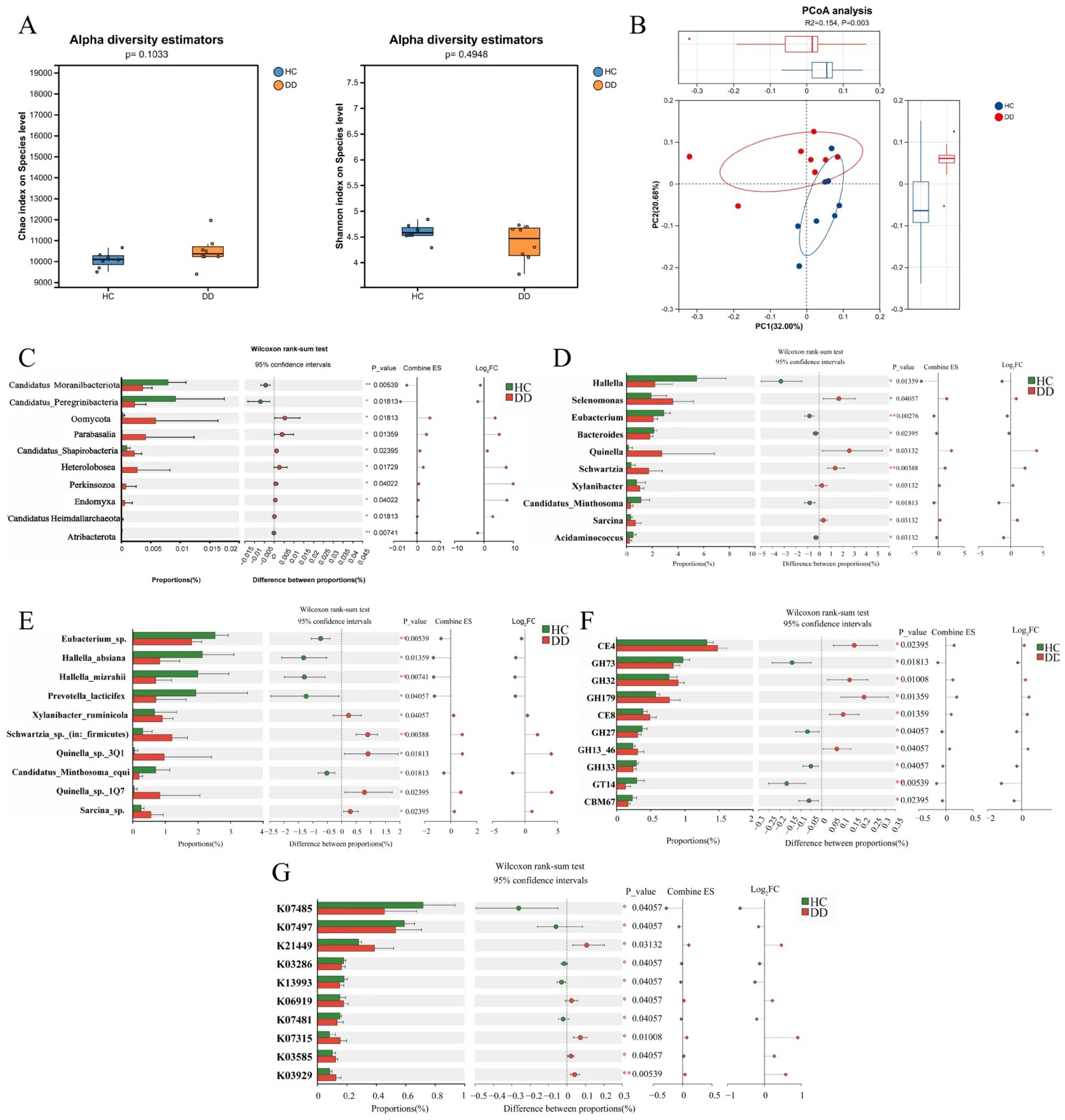
Effects of dietary DD supplementation on the structure and composition of the rumen microbial community in lambs. Analysis of the alpha and beta diversity via (**A**) Chao1 index and Shannon index, (**B**) principal coordinates analysis (PCoA) respectively. Microbial taxa differences between the HC and DD groups at the phylum (**C**), genus (**D**), and species (**E**) level. Differences in class-level CAZyme between HC and DD lambs (**F**). Differences KEGG Orthology entries (KOs) between HC and DD in the rumen microbiome of lambs (**G**). n = 8 per group. * *p* ≤ 0.05, ** *p* ≤ 0.01.

All rumen metagenome sequencing samples yielded 1,153,036,102 raw reads, with 1,146,594,638 reads retained after quality control. On average, each sample had 72,064,756 ± 4,484,952 raw reads, of which 71,662,165 ± 4,470,408 reads were retained post-quality control. Supplementation of DD altered rumen microbial composition, though α-diversity indices (Chao index and Shannon index) were unaffected (*p* > 0.05, Figure 4A). PCoA revealed a clear separation between the HC and DD groups, which was further supported by the Adonis test (*R*^2^ = 0.154, *p* = 0.003, Figure 4B).

As shown in Figure S1, *Bacteroidota* (53.05% vs. 50.04%) and *Bacillota* (33.47% vs. 35.65%) were the predominant phyla in the HC and DD group. Compared to the HC group, an upward trend was observed in the relative abundances of *Oomycota*, *Parabasalia*, *Candidatus_Shapirobacteria*, *Heterolobosea*, *Perkinsozoa*, *Endomyxa*, and *Candidatus_Heimdallarchaeota* in the DD group, whereas a downward trend was observed in *Candidatus_Moraniibacteriota*, *Candidatus_Peregrinibacteria*, and *Atribacterota.* (raw *p* < 0.05, *q* > 0.05, Figure 4C). At the genus level, the relative abundances of *Selenomonas*, *Quinella*, *Schwartzia*, *Xylanibacter*, and *Sarcina* in the DD group of lambs showed an upward trend compared to the HC group. However, the relative abundances of *Hallella*, *Eubacterium*, *Bacteroides*, *Candidatus_Minthosoma*, and *Acidaminococcus* showed a downward trend (raw *p* < 0.05, *q* > 0.05, Figure 4D). At the species level, *Xylanibacter_ruminicola*, *Schwartzia_sp._(in:_firmicutes)*, *Quinella_sp._3Q1*, *Quinella_sp._1Q7*, and *Sarcina_sp.* showed a tendency toward higher abundance, whereas *Eubacterium_sp.*, *Hallella_absiana*, *Hallella_mizrahii*, and *Prevotella_lacticifex* showed a tendency toward lower abundance in the DD group (raw *p* < 0.05, *q* > 0.05, Figure 4E). Carbohydrate-active enzyme (CAZyme) analysis revealed an upward trend in the abundances of CE4, GH32, GH179, CE8, and GH13_46 modules, and a downward trend in GH73, GH27, GH133, GT14, and CBM67 modules in the DD group relative to the HC group (raw *p* < 0.05, *q* > 0.05, Figure 4F). KEGG orthologous (KO) functional annotation revealed that K21449, K06919, K07315, K03585, and K03929 showed an upward trend in the DD group, whereas K07485, K07497, K03286, K13993, and K07481 showed a downward trend (raw *p* < 0.05; *q* > 0.05, Figure 4G). Notably, all differences were based on raw *p*-values; after FDR correction, none of these features reached statistical significance (*q* > 0.05, Table S1).

## 4. Discussion

High-concentrate diets provide a large amount of easily fermentable carbohydrates, which improve feed conversion efficiency. They are commonly used in lamb fattening to meet the various nutritional requirements necessary to maintain productivity [14]. Growth performance is a key economic indicator in lamb fattening, and appropriate nutritional management is crucial to improving production efficiency [15,16]. Findings from this experiment indicate that the addition of DD to a high-concentrate diet significantly increased the final BW, ADG and ADFI of lambs, while no significant difference was observed in F/G; One possible explanation is that dandelion powder may have enhanced diet palatability, thereby stimulating voluntary feed intake [17]. Growth and forage intake in ruminants are highly dependent on the condition of the rumen [18]; its histomorphology forms the structural basis for digestion and absorption, and changes in its morphology can improve growth performance in ruminants by enhancing nutrient transport across the ruminal epithelium [19]. Structure of the rumen wall, from inner to outer layers, consists of the epithelial cell layer, the basement membrane, the lamina propria and the muscular layer. Among these parameters, the length and width of the ruminal papillae and the thickness of the rumen wall are key indicators for assessing the morphological development of the rumen [20]. DD caused significant morphological changes in the rumen mucosa of lambs; nevertheless, these morphological differences were limited to a significant increase in papillae length, with no significant differences observed in the thickness of the epithelial cell layer, basement membrane, lamina propria or muscular layer. We speculate that DD improves rumen function primarily by promoting the development of rumen papillae. This may also be due to the influence of the active flavonoid compounds in DD on rumen development. Al-Ghamdi et al. reported that lambs of the Awasi breed fed a diet supplemented with citrus flavonoids exhibited greater ruminal papillae length, width, surface area and total surface area than lambs in the control group [21]. In contrast, the present study found that dandelion supplementation significantly increased only papilla length. This discrepancy may be attributed to differences in the forms and concentrations of bioactive compounds. The citrus flavonoid extract used by Ghamdi et al. was a standardized product with concentrated active ingredients, allowing for more direct and potent effects on ruminal epithelial proliferation and antioxidant status. In contrast, the 2 g/kg dandelion powder in the present study provided only approximately 70.0 mg/kg flavonoids, 87.0 mg/kg polyphenols, and 387.9 mg/kg polysaccharides—a substantially lower and qualitatively different bioactive profile that may compromise its bioavailability and bioactivity. Furthermore, breed-specific differences in ruminal baseline morphology and physiological responsiveness between Awassi and Dumeng crossbred lambs may also modulate the efficacy of phytogenic additives.

Excessive amounts of non-structural carbohydrates accelerate the rate of ruminal fermentation, leading to a significant accumulation of organic acids and volatile fatty acids, which can easily induce subacute ruminal acidosis in ruminants (SARA) [22]. The present study revealed that lambs in the HC group had a ruminal pH of 5.16, demonstrating a significant acidic environment under high-concentrate feeding, whereas the DD group showed a slightly higher pH without a statistically significant difference. There is a strong correlation between the sustained accumulation of VFA and the disruption of ruminal fermentation homeostasis [23]. Acetate, propionate, and butyrate constitute the major fraction of ruminal VFA, and their concentrations and relative proportions are widely used to characterize ruminal fermentation profiles [24]. The elevated VFA concentrations in the DD group are likely attributable to increased substrate availability for ruminal fermentation. Dandelion powder contains 193.93 mg/g of polysaccharides, which may provide additional fermentable carbohydrates for ruminal microorganisms, thereby promoting the production of acetate, propionate, and total VFA. This interpretation is supported by previous reports that phytogenic additives rich in polysaccharides and other carbohydrate fractions can enhance ruminal fermentation and increase VFA output [25,26,27]. Notably, the DD group exhibited significantly higher concentrations of acetate, propionate, valerate, and total VFA compared with the HC group, despite the lack of a corresponding pH increase. The significantly elongated ruminal papillae in the DD group suggest enhanced absorptive capacity, which may have facilitated VFA removal from the rumen lumen and partially offset the acidifying effect of VFA accumulation [28]. However, this interpretation remains tentative, owing to the absence of direct measurements for VFA absorption. In addition, the VFA composition differed between groups, with acetate and propionate significantly elevated in the DD group, whereas butyrate did not change significantly. Butyrate serves as the preferential energy substrate for ruminal epithelium and is rapidly metabolized upon absorption [29]. If epithelial absorptive function was indeed strengthened by dandelion supplementation, luminal butyrate would be efficiently absorbed. While acetate and propionate concentrations were markedly elevated in the DD group, no intergroup disparity was detected for butyrate levels, an observation that aligns with the above inference and indirectly supports the hypothesis of enhanced epithelial VFA absorption.

Notably, the acetate-to-propionate ratio did not differ between groups, indicating that the core fermentation pattern remained unaltered. No significant intergroup differences were detected for isobutyrate and isovalerate concentrations across treatments. These branched-chain VFAs are primarily derived from the catabolism of branched-chain amino acids and the turnover of ruminal microbial protein [30]. Ammonia nitrogen concentration in rumen fluid is an important indicator of microbial nitrogen utilization efficiency [31]. This study found that the addition of DD to the diet did not significantly alter the NH_3_-N concentration in the rumen of lambs, but it did significantly increase the total amino acid content in the rumen. It is speculated that this is due to the active compounds in DD inhibiting the activity of ruminal microbial deaminases and reducing the further breakdown of amino acids into ammonia, thereby redirecting nitrogen metabolism toward amino acid accumulation [32]. Studies have shown that adding quebracho extract to feed reduces protein deamination [33]. Cardozo et al. supplemented the diets of Holstein cows with a mixture of cinnamaldehyde and eugenol and found that it significantly inhibited deamination, as evidenced by increased concentrations of peptides and amino acids and decreased concentrations of NH_3_-N [34]. This is consistent with the results of this study. Collectively, these results indicate that dandelion supplementation altered ruminal fermentation and nitrogen metabolism in lambs fed a high-concentrate diet under the present experimental conditions.

As is well known, LPS is a potent pro-inflammatory endotoxin that triggers a systemic inflammatory response once it enters the bloodstream [35]. HIS is a key inflammatory mediator in ruminal acidosis; its excessive accumulation can damage the ruminal epithelium and disrupt tight junctions, and it can also synergize with LPS to amplify inflammation, exacerbating both local and systemic pathological damage [36]. In the present study, the DD group exhibited lower LPS and HIS levels together with decreased IL-1β and higher IL-10 concentrations. These coordinated changes suggest mild shifts in the local inflammatory environment. The positive correlation between LPS and IL-1β, together with the negative correlation between IL-10 and both LPS and IL-1β, suggests that the observed changes in these markers were interrelated. Dandelion extract has been shown to possess strong anti-inflammatory properties [37]. Dandelion polysaccharides can treat LPS-induced acute inflammation by modulating immune factors [38]. Studies have shown that supplementation with functional polysaccharides effectively reduces the levels of harmful metabolites—such as lactate, endotoxins, and histamine—induced by SARA, thereby alleviating inflammatory responses and epithelial damage [39]. The present findings are broadly consistent with these previous observations.

As an inflammatory trigger, LPS may also activate the classical TLR4-NF-κB inflammatory signaling pathway [40]. Wang et al. found that SARA increases the mRNA expression of TLR4 in the NF-κB inflammatory signaling pathway in rumen epithelium, thereby inducing rumen inflammation [8]. MyD88 is a key adaptor protein in the TLR4 signaling pathway that activates downstream NF-κB transcription factors [40]. In the present study, DD supplementation significantly reduced the mRNA expression of MyD88, whereas the mRNA levels of TLR4 and p65 were not significantly altered. This expression pattern implies that the canonical TLR4-MyD88-NF-κB cascade may not fully explain the observed transcriptional changes, suggesting that other regulatory pathways could also contribute to these molecular alterations.

Existing literature has documented that diets with elevated concentrate proportions can induce impaired mitochondrial function within ruminal epithelium and initiate whole-body inflammatory cascades [41]. HK-II is a key regulator of mitochondrial homeostasis [42], and VDAC1 oligomerization has been implicated as an upstream event in NLRP3 inflammasome activation following mitochondrial dysfunction [43]. Baik et al. demonstrated that oligomerized VDAC facilitates the aggregation of NLRP3 protein and triggers downstream inflammasome signaling [44]. After cellular stimulation, NLRP3 assembles with its adaptor ASC and pro-caspase-1 to construct intact inflammasome assemblies; this molecular assembly then drives caspase-1 maturation, which further cleaves precursor IL-1β and IL-18 into bioactive secreted isoforms [45]. Notably, a recent study in lactating Hu sheep demonstrated that high-concentrate feeding activated the mitophagy-NLRP3 inflammasome pathway in the liver, providing species-specific evidence that this pathway can be modulated by high-concentrate feeding in sheep, although direct evidence in ovine ruminal epithelial tissue remains limited [46]. In the present study, DD supplementation significantly upregulated HK-II mRNA and downregulated VDAC1, IP3R, NLRP3, and caspase-1 mRNA. At the same time, the decrease in IL-1β concentration was in the same direction as the decline in the expression levels of NLRP3 and caspase-1 mRNA. Flavonoids have long been proven to possess anti-inflammatory properties. Luteolin reduced the mRNA expression of Asc and Casp1, components of the NLRP3 inflammasome, in the adipose tissue of mice fed a high-fat diet, and decreased IL-1β secretion [47]. This is consistent with our findings. These coordinated transcriptional changes point toward the possibility that dandelion may modulate mitochondrial–endoplasmic reticulum interactions and potentially affect NLRP3 inflammasome-associated signaling in the lamb rumen.

As a glycolytic rate-limiting enzyme, HK-II drives glycolytic reprogramming to supply cellular energy [48], thereby supporting endothelial cell viability and ensuring adequate ATP for the synthesis and maintenance of tight junction proteins, which contributes to epithelial barrier integrity. Studies have reported that high-concentrate feeding can impair ruminal papillary morphology and disrupt the expression of tight junction-associated genes, compromising the structural integrity of the ruminal epithelium [49]. Tight junctions between epithelial cells form a continuous barrier that is essential for compartmentalizing tissue spaces and regulating the selective transport of solutes [50]. The tight junction complex is functionally defined by its core proteins, including occludin, claudins, and ZO-1 [51]. Steele et al. showed that under high-concentrate feeding conditions, the expression of genes encoding tight junction proteins in ruminal tissue is significantly downregulated [28]. Significant upregulation of ZO-1, occludin, and claudin-1 mRNA was observed in the DD group, which may contribute to the maintenance of ruminal epithelial barrier function. Changle et al. reported that baicalin treatment reduced TLR4, MyD88, p-NF-κB, and NLRP3 protein expression in LPS-challenged epithelial cells and attenuated epithelial injury [52].

Reportedly, the rumen microbiome is crucial for rumen health. To further investigate the regulatory effects of DD on the rumen, this study employed rumen metagenomic sequencing to analyze changes in the structure and species composition of the rumen microbiota under DD intervention. By combining α-diversity and PCoA clustering analysis, we identified overall community differences and simultaneously elucidated the expression patterns of functional modules encoding carbohydrate-active enzymes. PCoA analysis revealed statistically significant differences in bacterial composition between the two groups, a finding further supported by the Adonis test. Interestingly, while the DD supplementation group altered the rumen microbial composition, the α-diversity index remained unaffected. This is consistent with the findings reported by Luo [53]. Across all taxonomic groups, the *Bacteroidota* phylum accounted for the largest proportion of the two groups, followed by the *Bacillota* phylum. These two phyla accounted for nearly 85% of all sequences.

Although none of the observed differences at phylum, genus, or species levels survived FDR correction, several taxa with known functional relevance showed consistent directional trends at the raw *p* < 0.05 level and are discussed below. At the genus level, lambs in the DD group tended to have a higher relative abundance of *Selenomonas*, *Quinella*, *Schwartzia*, *Xylanibacter*, and *Sarcina* compared to lambs in the HC group. However, DD treatment tended to decrease the relative abundance of *Hallella*, *Eubacterium*, *Bacteroides*, *Candidatus_Minthosoma*, and *Acidaminococcus*. Wang et al. found that a compound of traditional Chinese herbs, whose primary active ingredients are flavonoids and flavonoid derivatives, can significantly increase the relative abundance of Selenomonas in the rumen of lambs [25]. *Quinella* is a unique, uncultured core bacterium found exclusively in the rumen. It metabolizes excess lactic acid in the rumen and promotes propionic acid synthesis, effectively preventing lactic acid accumulation and improving ruminal fermentation homeostasis [54]. In this study, the abundance of *Quinella_sp._3Q1* and *Quinella_sp._1Q7* showed an increasing trend following DD treatment. Similarly, the addition of Allium mongolicum Regel ethanol extract also increased the relative abundance of *Quinella* in the lamb rumen [55]. Research has shown that feeding mulberry leaf silage can modulate the immune system and rumen microbiota in lambs, and that Schwartzia expression is negatively correlated with the pro-inflammatory cytokine IL-6 [56]. This suggests that DD treatment may regulate the relative abundance of Schwartzia sp., potentially through anti-inflammatory mechanisms. *Xylanibacter* efficiently degrades various plant polysaccharides, including xylans, glucans, starch, β-glucans, and pectin [57]. It primarily produces propionic acid via the succinate pathway and serves as a key propionic acid-producing bacterium in the rumen [58], playing a central role in maintaining stable pH levels, promoting fiber digestion, and supplying energy. Following DD supplementation, the relative abundance of Xylanibacter ruminicola showed an upward trend, suggesting that DD may be associated with enhanced ruminal degradation and utilization of dietary polysaccharides. This finding aligns with the improved growth performance observed in the DD group. Mao et al. found that a high-concentrate diet significantly reduced the relative abundance of *Sarcina* [59]. In our study, the relative abundance of *Sarcina* sp. showed an upward trend following DD treatment. *Hallella* is a strictly anaerobic bacterium capable of fermenting carbohydrates and proteins; it primarily produces organic acids such as acetic acid and succinic acid, and is relatively sensitive to acidic environments [60]. In this study, the abundance of *Hallella absiana* and *Hallella mizrahii* showed a downward trend in the DD group, suggesting a potential decrease in organic acid production in the rumen. Mu et al. found that the relative abundance of Eubacterium was higher in the rumens of dairy cows fed a high-concentrate diet [61]. The DD treatment was associated with a decreasing trend in the relative abundance of both *Eubacterium* sp. and *Candidatus Minthosoma*. This finding is consistent with the study by Lu et al., in which the supplementation of goat feed with purple corn anthocyanins (phenolic compounds) similarly reduced the relative abundance of *Candidatus* in the rumen [62]. *Prevotella* species typically possess a high capacity for carbohydrate degradation and are closely associated with propionate fermentation. This genus can ferment a variety of substrates, including proteins, peptides, and hemicellulose, and is the primary producer of propionate in the rumen [63]. The downward trend in *Prevotella lacticifex* in the DD group may reflect altered fermentation dynamics toward a less acidogenic profile. Collectively, these compositional changes indicate that DD supplementation modulated ruminal microbial community structure and functional potential under high-concentrate feeding conditions.

Ruminants rely on a diverse array of polysaccharide hydrolases derived from ruminal microbiota to degrade plant biomass [64]. Among the enzyme families showing upward trends, both CE4 and CE8 are carbohydrate esterases that primarily participate in the deacetylation and demethylation of plant cell wall polysaccharides such as pectin and hemicellulose, exposing the polysaccharide backbone to facilitate subsequent degradation by glycosidase hydrolysis. These changes suggest that DD supplementation may enhance hemicellulose and pectin degradation by rumen microbiota, potentially contributing to improved rumen fermentation and growth performance in lambs [65,66,67]. Glycoside hydrolases (GH) are enzymes capable to hydrolyze the glycosidic bond between two carbohydrates or even between a carbohydrate and a non-carbohydrate moiety [68]. GH32 primarily encodes fructanase, sucrase, and other enzymes responsible for the catabolism of soluble carbohydrates such as fructans and sucrose [69], GH13_46 belongs to the α-amylase family and is involved in the ordered hydrolysis of starch and glycogen [70], their increased abundance helps reduce the excessive accumulation of soluble carbohydrates, alleviates abnormal fermentation and excessive lactic acid production, and thereby contribute to the maintenance of ruminal fermentation homeostasis. Currently, research on GH179, a glycosidase involved in polysaccharide degradation, is limited [71]. Among the enzyme families showing downward trends, GH73 is primarily involved in the metabolism of peptidoglycan and glucan in bacterial cell walls [72], and is often highly expressed during environmental stress or cellular damage [73]. Compared with the HC group, the downward trend in this gene in DD lambs suggests that dandelion supplementation may be associated with beneficial effects on ruminal microbial physiology under high-concentrate feeding conditions. GH27 is an α-galactosidase that plays a role in the metabolism of polysaccharides such as galactomannan and raffinose. This downward trend may indicate that DD is associated with altered microbial metabolic flux, with a trend toward reduced metabolism of related polysaccharides and upward trends in fiber degradation and propionate production pathways. GT14 is involved in carbohydrate synthesis [74], while CBM67 is primarily responsible for substrate binding [75]. Their downward trends may reflect a metabolic shift toward more efficient substrate utilization with lower energy expenditure. Collectively, these CAZyme trends suggest a potential shift in the carbohydrate-degrading capacity of the rumen microbiota, with possible enhancement of fiber degradation and propionate production and a reduction in energy consumption associated with polysaccharide biosynthesis and stress responses.

KO functional annotation analysis revealed trends in functional gene abundance that differed between the HC and DD groups, suggesting a possible association between dandelion supplementation and altered microbial metabolic profiles. Compared with the HC group, the abundance of functional genes associated with glycolysis and LPS biosynthesis (K07485, K07497, K03286, K13993, K07481) showed a decreasing trend in the DD group, whilst the abundance of functional genes associated with the tricarboxylic acid cycle, propionate synthesis, antioxidant defense and bile acid metabolism (K21449, K06919, K07315, K03585, K03929) showed an increasing trend. Previous studies have confirmed that excessive activation of the glycolytic pathway by microorganisms leads to a significant accumulation of lactic acid in the rumen, whilst abnormal enrichment of the LPS synthesis pathway results in the release of large amounts of endotoxins. Once these enter the bloodstream via the ruminal epithelium, they activate the body’s innate inflammatory response, serving as a major contributing factor to the epithelial damage associated with ruminal acidosis [76]. Conversely, the enrichment of pathways such as propionate and antioxidant metabolism can optimize ruminal acid–base homeostasis, clear excess oxidative products, and alleviate oxidative stress damage associated with high-concentrate feeding [77]. In this study, the abundance of harmful functional groups showed a downward trend whilst beneficial metabolic groups showed an upward trend, suggesting that DD supplementation was associated with altered metabolic functions at the microbial community level.

## 5. Conclusions

In summary, under the high-concentrate feeding conditions of the present study, dietary supplementation with dandelion improved growth performance, modulated ruminal fermentation and microbial community, and reduced inflammatory markers in lambs. These findings indicate a modulatory effect of dandelion on ruminal physiology. Given the short experimental duration, long-term effects and optimal inclusion level warrant further investigation. In addition, mRNA changes in NLRP3-related signaling suggest a potential mechanism requiring protein-level confirmation.

## Figures and Tables

**Table 1:** Composition and Nutritional Components of the Basal Diet (%, air-dry basis).

Ingredients	Content (%)	Nutrient Levels ^3^	Content (%)
Maize grain	39.00	Dry matter	90.60
Sunflower seed husks	22.00	Crude protein	14.12
Corn bran	10.00	Neutral detergent fiber	37.98
Soybean meal	8.00	Acid detergent fiber	16.05
Cottonseed meal	7.00	Crude ash	6.30
DDGS ^1^	5.50	Crude fat	2.10
Wheat bran	3.50	Calcium	1.01
Limestone powder	2.50	Phosphorus	0.27
NaCl	1.00	Metabolizable energy (MJ/kg)	10.59
Zeolite powder	0.78		
NaHCO_3_	0.30		
CaHPO_4_	0.30		
Vitamin-mineral premix ^2^	0.12		
Total	100.00		

Note. ^1^ DDGS stands for Corn Distillers Dried Grains with Solubles. ^2^ The premix provided the following per kg of diets: VA, 50,000 IU; VD, 315,000 IU; VE, 500 mg; Fe, 62.5 mg; Cu 12.5 mg; Zn, 125 mg; Mn, 62.5 mg; I, 1.875 mg; Se, 0.525 mg; Co, 0.5625 mg. ^3^ Metabolic energy is a calculated value; all other values are measured values.

**Table 2:** The primers information of genes in this study.

Gene	NucleotideSequences (5′ to 3′)	Size/bp	GenBank No.
ZO-1	F:AACCATCACGCCAGCATAC	209	ID: 443200
	R:CCACGCCACTGTCAAACTC		
Occludin	F:GGAGGAAGTGCCTTTGGTAG	100	ID: 101118304
	R:CCTTTGCCGCTCTTGGAT		
Claudin-1	F:GCTTCATCCTGGCGTTTC	126	ID: 780473
	R:TCCACAGCCCCTCGTAGA		
TLR4	F:AAAGAACTTGGAGGAGGGC	136	ID: 554263
	R:GGACACCACGACAATCACC		
MyD88	F:CGAGGAGGACTGCCAAAA	262	ID: 678682
	R:ACGGTCAGACACGCACAAT		
ASC	F:ACAGTCGTGGATGAGGTGC	232	ID: 101105208
	R:AGCCATTGCTGGAGAAGG		
HK-II	F:AGAACAAAGGCGTGGACC	137	ID: 101107690
	R:GCGGAGGAAGCGGACA		
VDAC1	F:AGGAAACAGCAACACTCGC	124	ID: 100145866
	R:TGGCTTTAGGGTCTGGGTA		
IP3R	F:TCTACTACCTGGGCATTGGG	275	ID: 114109674
	R:GCGGGTCACGCTCTTG		
p65	F:CGCAAAAGGACATACGAGAC	158	ID: 101102191
	R:GATGGCGTAAAGGAATAGGG		
NLRP3	F:CTCCATAACTCGCCCAAGG	278	ID: 101107699
	R:TGTCCCAACCACAATCTACG		
caspase-1	F:TCGGGCTGGCATTTGT	162	ID: 101117013
	R:ACCACCCCTTGCTTCTCA		

**Table 3:** Effects of DD on the growth performance of lambs.

Items	HC ^1^	DD ^2^	SEM ^3^	*p*-Value
Initial BW (kg)	31.70	32.40	0.43	0.43
Final BW (kg)	43.27	47.99	1.22	0.05
ADG (g/d)	192.72	259.76	16.04	0.03
ADFI (g/d)	1478.80	1997.24	110.64	0.01
F:G ratio	8.35	7.90	0.53	0.68

Note. ^1^ The high-concentrate diet group (HC): the ratio of concentrate to forage was 80:20. ^2^ The high concentrate diet supplemented with dandelion (DD): a basal diet (the ratio of concentrate to forage was 80:20) supplemented with 2 g/kg of dandelion. ^3^ SEM = standard error of the mean.

**Table 4:** Effects of DD on the ruminal fermentation parameters of lambs.

Items	HC	DD	SEM	*p*-Value
pH	5.16	5.36	0.10	0.30
NH_3_-N (mg/dL)	17.91	20.14	1.19	0.37
Total amino acid (umol/L)	16.13	35.37	3.15	<0.01
Total VFA (mmol/L)	110.33	166.25	11.13	0.01
**Individual VFA** (mmol/L)				
Acetate	57.80	82.66	5.19	0.01
Propionate	39.53	65.36	5.28	0.01
Butyrate	9.45	13.56	1.32	0.12
Isobutyrate	0.57	0.54	0.07	0.81
Valerate	2.10	3.00	0.19	0.01
Isovalerate	0.87	1.13	0.14	0.39
Acetate: Propionate	1.58	1.28	0.11	0.21

## Data Availability

The metagenomic sequencing data generated in this study have been deposited in the NCBI Sequence Read Archive (SRA) under BioProject accession number PRJNA1479742 and are publicly available. Other datasets used and analyzed during the current study are available from the corresponding author upon reasonable request.

## Supplementary Materials

The following supporting information can be downloaded at https://www.mdpi.com/article/10.3390/ani16162585/s1. Figure S1: Relative abundance of rumen microbiota at the phylum level; Table S1: Raw *p*-values and FDR-adjusted q-values for all taxon, CAZyme, and KO comparisons.
