# Supplementary material for: Effects of Dietary Dandelion Supplementation on Ruminal Morphology, Fermentation, Microbiome, and Inflammation in Lambs Under High-Concentrate Feeding
Source: Animals (Basel). 2026 Aug 19;16(16):2585. doi: 10.3390/ani16162585 (PMC13508958; doi:10.3390/ani16162585)
Supplement: Supplementary file 1 [file animals-16-02585-s001.zip › Figure S1.pdf]

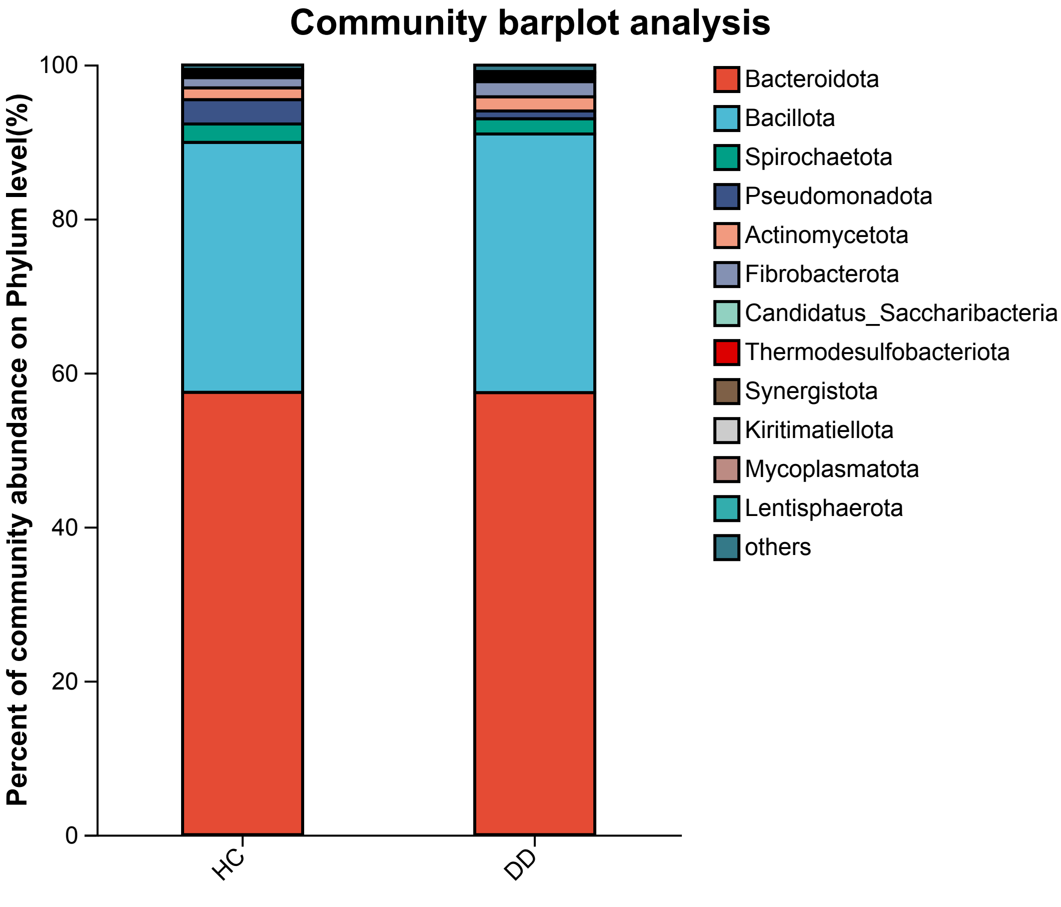
Community barplot analysis
Percent of community abundance on Phylum level(%)
100
80
60
40
20
0
HC
DD
Bacteroidota
Bacillota
Spirochaetota
Pseudomonadota
Actinomycetota
Fibrobacterota
Candidatus_Saccharibacteria
Thermodesulfobacteriota
Synergistota
Kiritimatiellota
Mycoplasmatota
Lentisphaerota
others
